# A new era for oral peptides: SNAC and the development of oral semaglutide for the treatment of type 2 diabetes

**DOI:** 10.1007/s11154-022-09735-8

**Published:** 2022-07-15

**Authors:** Vanita R. Aroda, Lawrence Blonde, Richard E. Pratley

**Affiliations:** 1 Brigham and Women’s Hospital, Harvard Medical School, Boston, MA USA; 2 Endocrinology Department, Ochsner Health, New Orleans, LA USA; 3 AdventHealth Translational Research Institute, Orlando, FL USA

**Keywords:** Sodium N-(8-[2-hydroxybenzoyl]amino)caprylate, SNAC, Semaglutide, Glucagon-like peptide-1 receptor agonists, GLP-1RA, Type 2 diabetes, Oral, Peptides

## Abstract

Glucagon-like peptide-1 (GLP-1) receptor agonists (GLP-1RAs) were first introduced for the treatment of type 2 diabetes (T2D) in 2005. Despite the high efficacy and other benefits of GLP-1RAs, their uptake was initially limited by the fact that they could only be administered by injection. Semaglutide is a human GLP-1 analog that has been shown to significantly improve glycemic control and reduce body weight, in addition to improving cardiovascular outcomes, in patients with T2D. First approved as a once-weekly subcutaneous injection, semaglutide was considered an ideal peptide candidate for oral delivery with a permeation enhancer on account of its low molecular weight, long half-life, and high potency. An oral formulation of semaglutide was therefore developed by co-formulating semaglutide with sodium N-(8-[2-hydroxybenzoyl]amino)caprylate, a well-characterized transcellular permeation enhancer, to produce the first orally administered GLP-1RA. Pharmacokinetic analysis showed that stable steady-state concentrations could be achieved with once-daily dosing owing to the long half-life of oral semaglutide. Upper gastrointestinal disease and renal and hepatic impairment did not affect the pharmacokinetic profile. In the phase III PIONEER clinical trial program, oral semaglutide was shown to reduce glycated hemoglobin and body weight compared with placebo and active comparators in patients with T2D, with no new safety signals reported. Cardiovascular efficacy and safety are currently being assessed in a dedicated outcomes trial. The development of an oral GLP-1RA represents a significant milestone in the management of T2D, providing an additional efficacious treatment option for patients.

## Introduction

Peptide and protein therapeutics play an increasingly important role in the treatment of numerous diseases, but the development of oral peptide therapies remains an ongoing challenge [1–4]. Due to their poor bioavailability when administered orally, peptide therapies typically require injection, which can impact on adherence, particularly in chronic diseases where long-term treatment is needed [2, 5–7]. The barriers to delivery of peptides via the oral route that can limit bioavailability include conditions in the stomach as well as low permeability of the gastrointestinal (GI) wall to peptides [2, 5]. The previous lack of progress in the development of orally administered peptides is evidenced by the failure of repeated attempts to develop an oral formulation of insulin since its discovery in 1921 [8].

Glucagon-like peptide-1 (GLP-1) receptor agonists (GLP-1RAs) are a highly efficacious class of drugs for the treatment of patients with type 2 diabetes (T2D) [9, 10]. GLP-1RAs provide effective glucose control while promoting weight loss via effects on appetite and gastric emptying [11–14]. Furthermore, selected GLP-1RAs have been shown to reduce the risk of cardiovascular (CV) events in individuals with established/high risk of CV disease (CVD) [15–17]. These benefits are reflected in the clinical guidelines published by the American Diabetes Association (ADA) and the American Association of Clinical Endocrinologists/American College of Endocrinology (AACE/ACE), which recommend use of a GLP-1RA or a sodium-glucose co-transporter-2 inhibitor (SGLT2i) with demonstrated CV risk reduction in patients with established/high risk of atherosclerotic CVD (ASCVD), while considering patient-specific factors [15, 17].

**Fig. 1:**
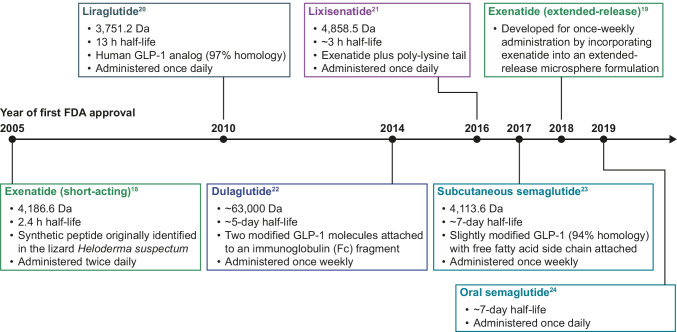
Summary of GLP-1RAs approved for the treatment of type 2 diabetes. *FDA* US Food and Drug Administration, *GLP-1* glucagon-like peptide-1, *GLP-1RA* glucagon-like peptide-1 receptor agonist

A number of GLP-1RAs are available for subcutaneous injection which differ in their molecular structure, size, half-life, and dosing interval (Fig. 1); these include exenatide (short-acting [18] and extended-release [19]), liraglutide [20], lixisenatide [21], dulaglutide [22], and semaglutide [23] (semaglutide is now also available in an oral formulation [24]). The first GLP-1RA to be approved was exenatide in 2005 [18]. Exenatide is a synthetic peptide analog of GLP-1 (4,186.6 Da), originally identified in the lizard *Heloderma suspectum* [18]. Exenatide (short-release) has a mean terminal half-life of 2.4 h and is administered twice daily [18]. An extended-release formulation of exenatide was subsequently developed for once-weekly administration by incorporating exenatide into an extended-release microsphere formulation [19]. Lixisenatide (4,858.5 Da) – an exenatide derivative – has a half-life of ~ 3 h and is suitable for once-daily administration [21, 25]. Liraglutide is a human GLP-1 analog (3,751.2 Da) with 97% homology to native GLP-1, designed to bind to albumin via a fatty acid and a spacer covalently attached to the peptide backbone [20]. Liraglutide has a half-life of 13 h and is also administered once daily [20].

Dulaglutide and subcutaneous semaglutide are longer-acting compounds that require once-weekly administration [22, 23]. Dulaglutide (~ 63,000 Da) is derived from two modified GLP-1 molecules attached to an Fc fragment of immunoglobulin G and has a half-life of ~ 5 days [22]. Semaglutide (4,113.6 Da) is a human GLP-1 analog, similar to liraglutide but engineered to have increased albumin affinity and resistance to dipeptidyl peptidase-4 (DPP-4) inhibitor degradation to permit once-weekly subcutaneous administration [12, 23]. The efficacy of subcutaneous semaglutide was established in the phase III SUSTAIN program (SUSTAIN 1–5 and 7), in which significant reductions in mean glycated hemoglobin (HbA_1c_) and body weight were demonstrated against placebo and a variety of active comparators in multiple patient groups with T2D [26–33]. Furthermore, in SUSTAIN 6, a dedicated CV outcomes trial (CVOT), a 26% reduction in major adverse CV events (MACE) was observed with subcutaneous semaglutide compared with placebo (p < 0.001 for non-inferiority; p = 0.02 for superiority, not prespecified) [30]. This led to the US Food and Drug Administration (FDA)-approved indication for subcutaneous semaglutide for reducing MACE in patients with T2D and CVD [23, 30].

Despite their importance, GLP-1RAs were, until recently, only available for subcutaneous administration, which may have contributed to lower usage rates versus orally available therapies (i.e., SGLT2is and DPP-4 inhibitors) [6, 7, 34–37]. In 2019, an oral formulation of semaglutide became the first orally administered GLP-1RA to be approved [3, 24]. It was developed by co-formulating semaglutide with the permeation enhancer sodium N-(8-[2-hydroxybenzoyl]amino)caprylate (SNAC) to overcome the challenges of oral peptide delivery [24, 38]. The addition of oral semaglutide expands the treatment options available to those with T2D and may encourage increased and earlier use of GLP-1RAs, thereby enabling more patients to achieve better glycemic control [37–39].

This review will examine the development of oral semaglutide, including the challenges that had to be overcome to produce the first oral peptide for T2D, other technologies developed to date, and the role of SNAC in the development of oral semaglutide. We will also review the mechanism by which SNAC enhances absorption of oral semaglutide and highlight the key preclinical and clinical data that led to approval of the first orally administered GLP-1RA for T2D.

## Challenges of developing an oral peptide therapy

The oral administration of peptides is not a new concept, and the challenges faced are best illustrated by considering the failure to date to produce a marketable oral formulation of insulin since its discovery in 1921 [1, 2, 8, 40]. The first attempt to deliver insulin orally was described in 1923 and involved the use of dilute alcohol as a solvent [41]; since then, there have been multiple other attempts, including some using permeation enhancers [6]. Several barriers must be overcome when developing orally administered peptides with a molecular weight above 1,000 Da, including enzymatic degradation in the GI tract, pH-induced conformational changes, limited permeability of the intestinal membrane, and variable GI tract absorption rates [2, 3, 42, 43]. To overcome these barriers, peptides ideally need to have a large therapeutic index, some stability in the GI tract, a long elimination half-life, and a relatively low clearance rate [3, 43].

A number of technologies for the oral delivery of peptides – typically insulin – have been described in the literature, including nanoparticles, microneedle devices, self-emulsifying drug delivery systems, peptide conjugation, and permeation enhancers (reviewed in detail by Durán-Lobato et al. [44] and Zizzari et al. [45]). Of these, permeation enhancers are the most widely tested approach to improve oral absorption of peptides, due to the relative ease with which they can be incorporated into formulations compared with nanotechnology or device-based systems [43, 46].

In recent years, an oral formulation of a long-acting basal insulin analog (IO338) with the permeation enhancer sodium caprate was shown in a phase II study to have comparable efficacy to subcutaneous insulin glargine [47]. While this demonstrated the utility of permeation enhancers, further development of IO338 was discontinued because the doses required were high and were judged not to be commercially viable [47].

## A brief history of SNAC

SNAC was developed in the 1990s by Emisphere (US) as part of a wider effort to identify carrier-based permeation enhancers that could chaperone candidate peptides across the gastric lining [43, 46]. SNAC is a synthetic N-acetylated amino-acid derivative of salicylic acid that displays amphiphilicity. It is a transcellular, carrier-based permeation enhancer that, unlike others, does not require a protective enteric coating [43].

Successful application of SNAC was first demonstrated by the approval of an oral formulation of vitamin B12 (cyanocobalamin/SNAC) in 2014 by the US FDA, albeit as a medical food [43, 48, 49]. As part of the approval process, SNAC was assigned generally regarded as safe (GRAS) status by the US FDA [43, 49].

SNAC has since been extensively tested with a range of poorly permeable molecules, with limited success. SNAC was first utilized to develop an oral formulation of unfractionated heparin that historically had required parenteral administration [43, 50–53]. In phase I/II studies, oral heparin/SNAC was shown to have similar activity to subcutaneous heparin, but in subsequent phase III trials it did not demonstrate superiority to subcutaneous enoxaparin [53, 54]. Further development of an orally administered heparin/SNAC soft gelatin capsule was later discontinued, possibly due to the introduction of other anti-thrombotics [43, 55].

In 2017, subcutaneous semaglutide was approved for once-weekly administration [23], becoming only the second approved once-weekly GLP-1RA (after dulaglutide). However, there remained an unmet need for an oral agent as an alternative option to subcutaneously administered GLP-1RAs. Semaglutide was an ideal candidate for oral delivery with SNAC due to its low molecular weight (4,113.6 Da), long half-life (~ 7 days after subcutaneous administration), and high potency relative to other peptides [14, 40, 46, 56]. SNAC was therefore co-formulated with semaglutide to produce the first GLP-1RA suitable for oral administration.

## Mode of action of SNAC in oral semaglutide: preclinical and early clinical studies

**Fig. 2:**
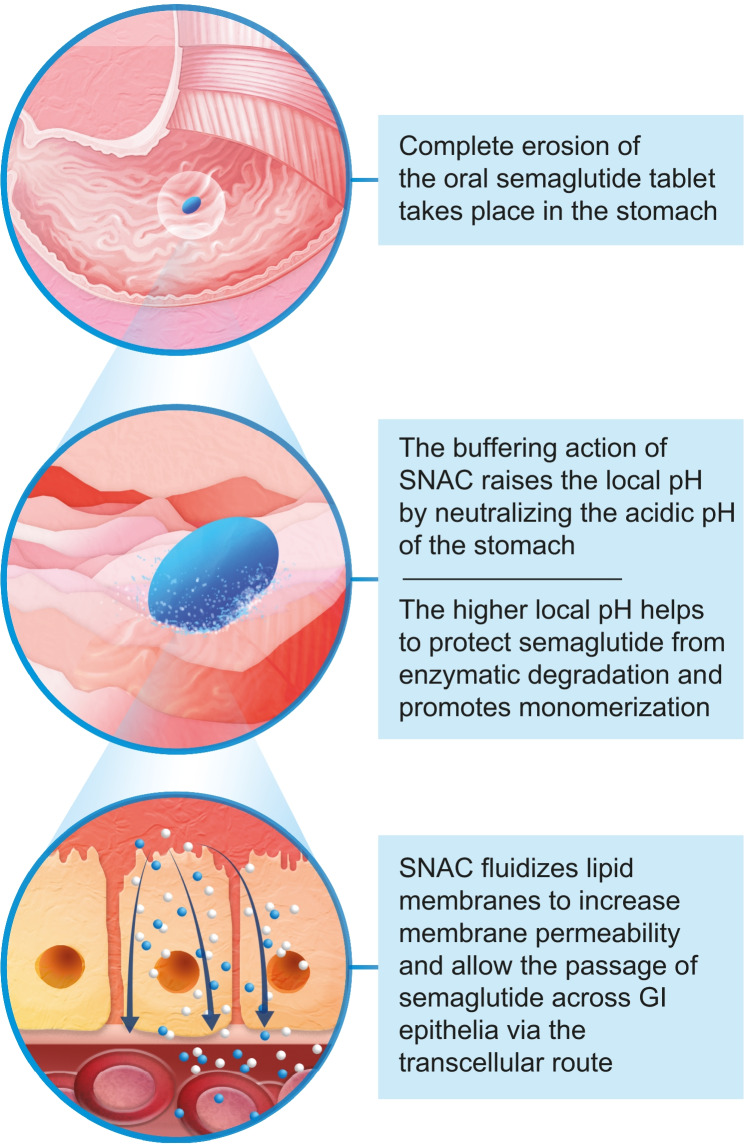
Mechanism of absorption and protection of the semaglutide molecule [38, 56].* GI* gastrointestinal, *SNAC* sodium N-(8-[2-hydroxybenzoyl]amino)caprylate

The proposed mechanism of action of SNAC when co-formulated with semaglutide is shown in Fig. 2 [56]. As the tablet is eroded, SNAC causes a local increase in pH via a buffering action. *In vitro* evidence suggests that this increase in gastric pH may protect semaglutide from enzymatic degradation by reducing the conversion of pepsinogen to pepsin [56]. In addition, SNAC promotes monomerization of semaglutide by changing the polarity of the solution in which the tablet dissolves, thereby weakening the hydrophobic interactions that would otherwise promote semaglutide oligomerization [56]. The enhanced absorption of semaglutide is thought to be due to the indirect action of SNAC, which is incorporated into the lipid membrane of local gastric cells and fluidizes the plasma membrane of the gastric epithelium (a solid-to-fluid structural transition), allowing transcellular passage of semaglutide [3, 56]. Mechanistic analyses suggest that this action is transient and fully reversible [56].

Findings from basic science and clinical research suggest that, in contrast to most oral drugs, semaglutide co-formulated with SNAC is absorbed in the stomach [56]. Scintigraphic imaging of human volunteers following a single dose of oral semaglutide (10 mg with 300 mg SNAC) demonstrated erosion of the tablet and absorption of semaglutide in the stomach [56]. In addition, plasma semaglutide levels were similar in dogs that had undergone pyloric ligation (to prevent intestinal absorption) compared with non-ligated dogs, and plasma concentrations in the splenic vein (draining the gastric cavity) were significantly higher than in the portal vein (draining the GI tract), further implicating the stomach as the site of absorption [56].

The absorption-enhancing action of SNAC is thought to be highly dependent on the specific agent it is enhancing, which means that carefully tailored co-formulation is required rather than co-administration [56]. The structure of liraglutide (a structurally distinct analog of GLP-1RA) was found to be unfavorable for co-formulation with SNAC on account of its stronger membrane-binding properties, which reduced transcellular passage, as well as its greater tendency to oligomerize, which countered the monomerizing effects of SNAC [56]. In a preclinical study, plasma exposure was significantly higher for semaglutide than liraglutide after oral dosing with SNAC [56].

## Early clinical development (phase I, phase II) of oral semaglutide

### Phase I: pharmacokinetic profile of oral semaglutide

#### Food and dosing conditions

**Fig. 3:**
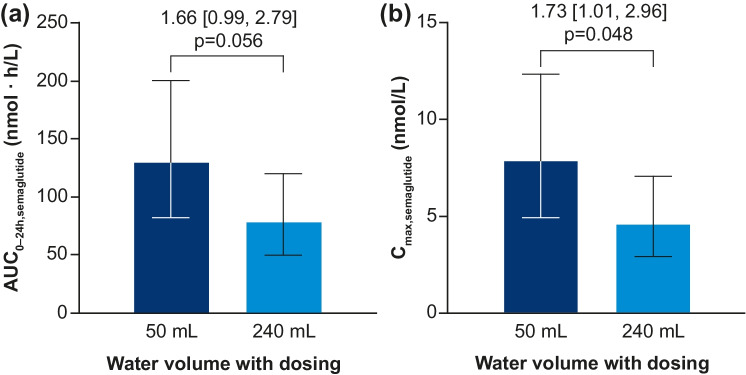
Effect of water volume with dosing on (**a**) AUC_0–24 h,semaglutide_ and (**b**) C_max,semaglutide_ after a single dose of 10 mg oral semaglutide in healthy male subjects [58]. Bars are estimated means and 95% CIs. Treatment comparisons show estimated treatment ratios (95% CI) and p-value. Endpoints were analyzed on a logarithmic scale but are presented on the linear scale. n = 24 (50 mL) or n = 26 (240 mL). Conversion factor from molar concentration (nmol/L) to mass concentration (ng/mL), 4.11358. Reprinted from: Bækdal et al. Clin Pharmacol Drug Dev. 2021;10(5):453–61. https://doi.org/10.1002/cpdd.938. ^©^2021 The Authors. With permission from Wiley Periodicals LLC on behalf of American College of Clinical Pharmacology (CC BY-NC-ND 4.0). *AUC* area under the curve, *CI* confidence interval, *C*_*max*_ maximum concentration

In phase I studies, food and drink were found to impact the absorption of oral semaglutide, with limited or no measurable absorption reported in fed participants [57]. Oral semaglutide absorption increased with longer post-dose fasting periods and was comparable when administered with either 50 or 120 mL of water [57]. Complete tablet erosion was observed regardless of water volume but occurred at a slower rate with 50 mL versus 240 mL, resulting in higher semaglutide plasma exposure with lower water volumes (Fig. 3) [58]. SNAC absorption was generally rapid and eliminated with no measurable exposure at ~ 4 to 6 h post-dose, while the terminal half-life of semaglutide (t_1/2,semaglutide,day 10_) was ~ 1 week, showing that, unlike initial absorption, the metabolism and elimination of semaglutide are not affected by food ingestion or water volume [57]. These findings informed the phase II and III study recommendations to administer oral semaglutide in the fasting state with up to 120 mL water and wait for 30 min post-dose before eating or ingesting other oral medications [39, 57, 59–62].

#### Effects on satiety

In an exploratory, phase I study, oral semaglutide was found to reduce hunger, cravings for high-fat foods, and calorie consumption in patients with T2D [63], consistent with results from an earlier study of subcutaneous semaglutide in patients with obesity [64]. These results suggest that semaglutide, like other GLP-1RAs, may contribute to promoting healthy lifestyle changes in addition to glycemic control.

### Phase II: dose selection of semaglutide and SNAC

Different combinations of semaglutide and SNAC doses were explored in phase I/II studies [59, 65]. In a phase II dose-finding study, oral semaglutide doses of 2.5 mg to 40 mg co-formulated with 300 mg SNAC improved glycemic control, with a dose-dependent mean reduction in HbA_1c_ of up to 1.9% versus 0.3% for placebo, from a mean HbA_1c_ level of 7.9% (standard deviation: 0.7%) [59]. The safety profile of oral semaglutide was consistent with the known adverse effects of other GLP-1RAs, and there were no unexpected safety findings [59]. The most common adverse events reported with oral semaglutide were GI events and were mostly mild to moderate in severity [59]. These adverse events were most frequent during the dose-escalation period and decreased over time with continued semaglutide treatment [59].

In a single-dose trial, semaglutide exposure was highest when administered with 300 mg of SNAC, compared with 150 mg or 600 mg [65]. In a multiple-dose study of semaglutide co-formulated with 300 mg SNAC, semaglutide exposure was two-fold higher with 40 mg versus 20 mg oral semaglutide, demonstrating dose proportionality [65]. The half-life of oral semaglutide was also ~ 1 week in both groups, which was comparable with subcutaneous semaglutide and illustrates that the half-life is not determined by the mode of administration [65]. No unexpected safety signals were observed; oral semaglutide up to 40 mg was safe and tolerable with the overall safety profile consistent with that of the GLP-1RA class [65]. Based on these results, oral semaglutide co-formulated with 300 mg of SNAC was selected for further development [65].

#### Bioavailability of orally administered semaglutide

Model-based analysis of semaglutide pharmacokinetics suggests that once absorbed, semaglutide is distributed, metabolized, and eliminated in the same way regardless of the administration route (intravenous, subcutaneous, or oral) [66]. A population pharmacokinetic model developed using data from clinical trials of subcutaneous and intravenous semaglutide was modified to include data from six trials of oral semaglutide in healthy volunteers or patients with T2D and renal or hepatic impairment [66, 67]. The bioavailability of semaglutide was 0.8% when using the recommended dosing conditions [66]. Bioavailability increased with a longer post-dose fasting time, reaching a plateau of 1.4% at around 120 min, and decreased with higher water volumes (240 mL) [66]. Within-subject variability of bioavailability was estimated to be high at 137%, but this was reduced to 33% in the steady state [66]. Bioavailability did not differ significantly between healthy participants and those with T2D [66]. Oral semaglutide was absorbed significantly faster, and showed lower and more variable bioavailability, than subcutaneous semaglutide [66]. However, based on model-derived conclusions, once-daily dosing and the long half-life of oral semaglutide reduced day-to-day variability, resulting in stable steady-state concentrations [66]. According to the label, steady-state exposure of oral semaglutide is achieved following 4–5 weeks’ administration [24].

#### Impact of comorbidities commonly seen in patients with T2D on oral semaglutide absorption

Upper GI disease and hepatic and renal impairment are common comorbidities in patients with T2D that may influence the pharmacokinetics of antidiabetes drugs [68, 69]. However, pharmacokinetic studies suggest that these comorbidities do not affect the pharmacokinetics of oral semaglutide, and based on the observed safety profiles, no dose adjustments are recommended [68, 69].

#### Interactions with drugs commonly administered in patients with T2D

Many individuals with T2D are required to take multiple medications [70]. Given the mechanism of action of oral semaglutide [56], which includes delayed gastric emptying [24], a number of studies have assessed the impact of oral semaglutide/SNAC on exposure to other commonly administered oral drugs [71–73]. In a pharmacokinetic study, co-administration of oral semaglutide with multiple (five) placebo tablets reduced the absorption of semaglutide [74]. Hence, clinical dosing guidance states that other oral medications should be administered at least 30 min after oral semaglutide [24, 74].

Other pharmacokinetic studies have shown no clinically relevant interactions – indicating that no dose adjustments are required – when oral semaglutide is administered in people receiving lisinopril, warfarin, metformin, digoxin, furosemide, rosuvastatin, ethinylestradiol/levonorgestrel, or omeprazole [24, 71–73]. In a study of healthy subjects, concomitant administration of oral semaglutide 2 h after intake of omeprazole – which increases gastric pH – led to a 14% increase in the area under the curve (AUC)_0–24 h,Day10_ of SNAC compared with administration of oral semaglutide alone, whereas SNAC maximum concentration and time to maximum concentration (t_max_) were similar with and without omeprazole. In most patients, plasma concentrations of SNAC fell below the lower level of quantification within 24 h, indicating rapid elimination [71]. Exposure to oral semaglutide was also slightly increased when administered with omeprazole compared with when administered alone (AUC_0–24 h,Day10_), but this difference was not statistically significant (estimated treatment ratio: 1.13; 90% confidence interval: 0.88, 1.45) and the median t_max_ and t_1/2_ for oral semaglutide were similar with and without omeprazole [71]. This slight increase in exposure to semaglutide with omeprazole was not considered to be clinically relevant and no dose adjustments are recommended [71].

Co-administration of oral semaglutide with 600 μg of levothyroxine – an oral medication with similar dosing requirements to semaglutide, namely administration once daily on an empty stomach with a full glass of water, 30–60 min before breakfast [24, 75] – increased exposure to thyroxine by 33% (AUC_0–48 h,T4_), supporting the recommendation to avoid co-administration of oral semaglutide within 30 min of other oral medications [24, 74]. No obvious effect on thyroxine exposure was seen with SNAC alone, indicating that the increased exposure may be due to the delayed gastric emptying effect of semaglutide [74].

In addition to advising patients to wait at least 30 min before taking other oral medications after oral semaglutide, increased clinical and laboratory monitoring should be considered when prescribing medications with a narrow therapeutic index or those that require clinical monitoring, such as levothyroxine [24, 75].

## Phase III: efficacy and safety of oral semaglutide in T2D

### Overview of the PIONEER program (PIONEER 1–8)

The efficacy and safety of oral semaglutide have been extensively evaluated across the continuum of T2D in the phase III PIONEER clinical trial program [39, 61, 62, 76–82]. PIONEER 1–8 enrolled patients with early, established, and advanced T2D from a global population. The details of the PIONEER program, including study design and results, have been published and reviewed extensively, therefore only a brief overview is provided here [38, 39, 61, 62, 76–82].

The PIONEER trials were among the first in T2D to utilize estimands based on regulatory guidelines during trial planning, trial conduct, data analysis, and interpretation of results [60]. Estimands are defined as targets of estimation based on the trial objectives, providing greater transparency of the effects reported [60]. The treatment policy estimand evaluated the effect of treatment regardless of treatment discontinuation or initiation of rescue medication [60], comparable to an intention-to-treat approach. The trial product estimand evaluated the treatment effect while on treatment and without the use of rescue medication [60], providing a more direct evaluation of medication efficacy. Further detail is provided by Aroda et al. [38, 60]. Throughout this review, the treatment policy estimand is reported unless otherwise specified.

### Key endpoints in the PIONEER program (PIONEER 1–8)

The primary endpoint in PIONEER 1–5 [39, 61, 62, 76, 77] and PIONEER 8 [82] was change from baseline in HbA_1c_, while change from baseline in body weight at Week 26 was included as a secondary endpoint. PIONEER 7 [81] was a flexible-dose study and PIONEER 6 was a CV safety trial [79]. Safety endpoints across all PIONEER studies included the number of treatment-emergent adverse events, hypoglycemic episodes, laboratory tests, physical examinations, and predefined outcomes of special interest [38, 39, 61, 62, 76–82].

### Patient population in the PIONEER program (PIONEER 1–8)

Across the PIONEER program, the majority of patients were adults (≥ 18 years) with a diagnosis of T2D and baseline HbA_1c_ levels in the range of 7.0–9.5% [38, 39, 61, 62, 76–82]. In PIONEER 6, patients were aged ≥ 50 years and had clinical evidence of CVD or chronic kidney disease, or were aged ≥ 60 years with CV risk factors [38, 79]. A total of 8,842 patients were randomized to receive oral semaglutide or comparators during the PIONEER program (1–8) and over 80% completed each trial [38, 39, 61, 62, 76–82].

### Doses and comparators in the global trials (PIONEER 1–8)

In the PIONEER studies, oral semaglutide doses of 14 mg, 7 mg, and in some cases, 3 mg, were tested against either placebo or active comparators [38, 39, 61, 62, 76–84]. PIONEER 1 compared oral semaglutide with placebo [76], while PIONEER 2–4 compared oral semaglutide with the SGLT2i empagliflozin (25 mg once daily), the DPP-4 inhibitor sitagliptin (100 mg once daily), and the subcutaneous GLP-1RA liraglutide (1.8 mg once daily) [38, 39, 61, 62]. In PIONEER 7, flexible dose adjustment of oral semaglutide was compared with sitagliptin (100 mg) [81], while in PIONEER 5, 6, and 8, patients received either oral semaglutide or placebo added to background medication [38].

### Key efficacy outcomes of the global trials (PIONEER 1–8)

**Fig. 4:**
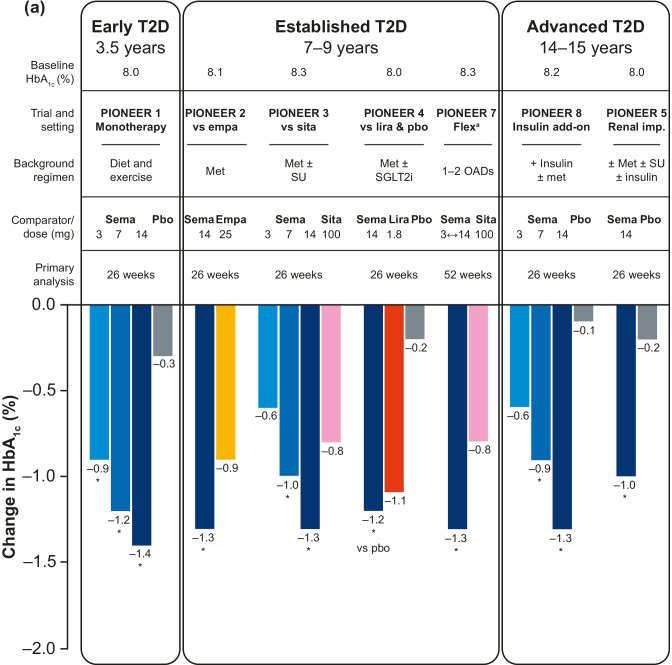

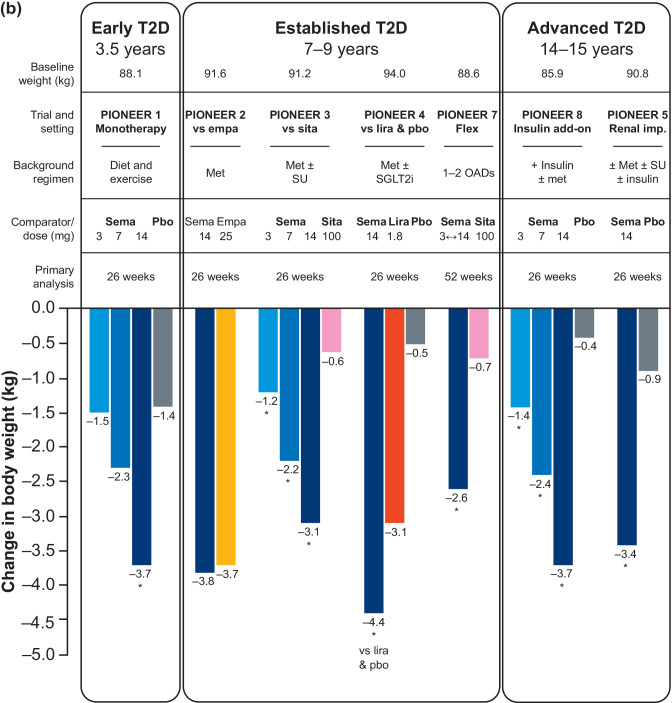
Key efficacy outcomes of the global PIONEER studies (PIONEER 1–5 and PIONEER 7–8). (**a**) Reduction in HbA_1c_ with oral semaglutide and comparators at the primary analysis time point (26 weeks except for PIONEER 7). Data are for the treatment policy estimand (including data from patients who discontinued treatment or required rescue medication). (**b**) Reduction in body weight with oral semaglutide and comparators (26 weeks except for PIONEER 7). Data are for the treatment policy estimand (including data from patients who discontinued treatment or required rescue medication). Reprinted from: Thethi et al. Diabetes Obes Metab. 2020;22(8):1263–77. https://doi.org/10.1111/dom.14054. ^©^2020 The Author(s). With permission from John Wiley & Sons Ltd (CC BY-NC 4.0). ^a^HbA_1c_ reduction was not the primary endpoint in PIONEER 7. *p < 0.05 for the estimated treatment difference with oral semaglutide versus placebo and/or active comparator. *empa* empagliflozin, *HbA*_*1c*_ glycated hemoglobin, *imp* impairment, *lira* liraglutide, *met* metformin, *OAD* oral antidiabetes drug, *pbo* placebo, *sema* semaglutide, *SGLT2i* sodium-glucose co-transporter-2 inhibitor, *sita* sitagliptin, *SU* sulfonylurea, *T2D* type 2 diabetes

The key efficacy outcomes of the global PIONEER studies (PIONEER 1–5 and PIONEER 7–8) are summarized in Fig. 4 [38].

#### Oral semaglutide monotherapy in early T2D (mean disease duration: 3.5 years)

In patients with T2D insufficiently controlled by diet and exercise alone (PIONEER 1), oral semaglutide significantly reduced HbA_1c_ versus placebo by an estimated treatment difference (ETD) of –0.6% (3 mg) to –1.1% (14 mg) from baseline at Week 26 (p < 0.001) [76]. Oral semaglutide also reduced body weight from baseline in a dose-dependent manner by an ETD between –0.1 kg (3 mg) and –2.3 kg (14 mg, p < 0.001) at Week 26 [76].

#### Oral semaglutide in established T2D (mean disease duration: 7–9 years)

In patients with T2D uncontrolled on metformin (PIONEER 2), oral semaglutide (14 mg) provided significantly greater reductions in HbA_1c_ versus empagliflozin (25 mg) at Week 26 (–1.3% vs –0.9%; ETD: –0.4%; p < 0.0001) [61]. Oral semaglutide was not superior to empagliflozin in change from baseline in body weight at Week 26 or Week 52 by treatment policy estimand but was significantly better at Week 52 (–4.7 vs –3.8 kg; p = 0.0114) by the trial product estimand [61]. In patients with T2D uncontrolled with metformin (with or without sulfonylurea) (PIONEER 3), oral semaglutide (7 and 14 mg) significantly reduced HbA_1c_ versus sitagliptin 100 mg (ETD: –0.3% and –0.5%, respectively; p < 0.001 for both) by the treatment policy estimand [62]. Oral semaglutide (7 and 14 mg) was superior to sitagliptin in reducing body weight from baseline at Week 26 (ETD: –1.6 kg and –2.5 kg, respectively; p < 0.001 for both) [62]. In patients with T2D uncontrolled on metformin with or without an SGLT2i (PIONEER 4), oral semaglutide was non-inferior to subcutaneous liraglutide in decreasing HbA_1c_ (ETD: −0.1%; p < 0.0001) and superior to placebo (ETD: –1.1%; p < 0.0001) at Week 26 [39]. Oral semaglutide also resulted in superior weight loss compared with liraglutide (ETD: –1.2 kg; p = 0.0003) and placebo (ETD: −3.8 kg; p < 0.0001) at Week 26 [39]. In PIONEER 7, flexible dose adjustment of oral semaglutide was more effective than sitagliptin 100 mg in reducing HbA_1c_ (ETD: –0.5%; p < 0.0001) at Week 52 (at which time 30% of patients in the oral semaglutide group were receiving the 7 mg dose and 59% the 14 mg dose) [81].

#### Oral semaglutide in advanced T2D (mean disease duration: 14–15 years)

In patients with long-standing T2D and moderate renal impairment (estimated glomerular filtration rate 30–59 mL/min/1.73 m^2^) (PIONEER 5), oral semaglutide 14 mg was significantly more effective than placebo in reducing HbA_1c_ (ETD: –0.8%; p < 0.0001) and body weight at Week 26 (ETD: –2.5 kg; p < 0.0001) [77]. In patients with T2D at high CV risk in PIONEER 6, oral semaglutide reduced HbA_1c_ compared with placebo as an add-on to standard of care (mean change of –1.0% vs −0.3%) at the end of the trial (not statistically analyzed; event-driven with follow-up continuing until accrual of ≥ 122 primary outcome events) [79].

In patients with advanced T2D uncontrolled on insulin with or without metformin (PIONEER 8), oral semaglutide significantly reduced HbA_1c_ compared with placebo at Week 26 (ETD: −0.5% [3 mg] to −1.2% [14 mg]; p < 0.0001 for all) [82]. Oral semaglutide was also superior to placebo in reducing body weight (ETD: –0.9 kg, p = 0.0392 [3 mg] to –3.3 kg, p < 0.0001 [14 mg]) at Week 26 [82].

##### Other outcomes of the global phase III trials

A greater proportion of patients consistently achieved the ADA-recommended glycemic target (HbA_1c_ < 7%) with oral semaglutide 7 and 14 mg (42–77%) versus placebo (7–31%) and active comparators (25–62%) at the primary analysis time point across the global trials (PIONEER 1–5 and PIONEER 7–8) with no weight gain or severe/blood glucose-confirmed hypoglycemia [38, 39, 61, 62, 76, 77, 81, 82]. Furthermore, a greater proportion of patients achieved HbA_1c_ reduction ≥ 1% with body weight loss ≥ 3% at Week 26 with oral semaglutide 7 and 14 mg than with placebo and active comparators [38, 39, 61, 62, 76, 77, 81, 82]. Quality-of-life outcomes were similar between oral semaglutide and active comparators (including empagliflozin, sitagliptin, and subcutaneous liraglutide) across the trials [38, 39, 61, 62, 76, 77, 81, 82].

Consistent with these data from the pivotal trials, preliminary evidence from studies of early adoption of oral semaglutide in the US also suggests real-world improvements in glycemic control, with a mean HbA_1c_ reduction of 0.9% observed across patients [85, 86]. The PIONEER REAL prospective study is currently enrolling patients as part of local clinical practice in Canada, Europe, and Japan [87–93].

##### Safety and tolerability

Across the PIONEER program, the safety and tolerability of oral semaglutide were generally consistent with the known profile of GLP-1RAs; the most frequent adverse events reported were GI disorders, mainly nausea, vomiting, diarrhea, constipation, dyspepsia, and upper abdominal pain [38]. In PIONEER 4, the safety and tolerability of oral semaglutide were consistent with those of subcutaneous semaglutide and with the GLP-1RA class in general [39]. Adverse events were slightly more frequent with oral semaglutide than with subcutaneous liraglutide [39]. However, the majority of adverse events in both treatment groups were mild-to-moderate GI events, with the most common being transient nausea [39].

**Fig. 5:**
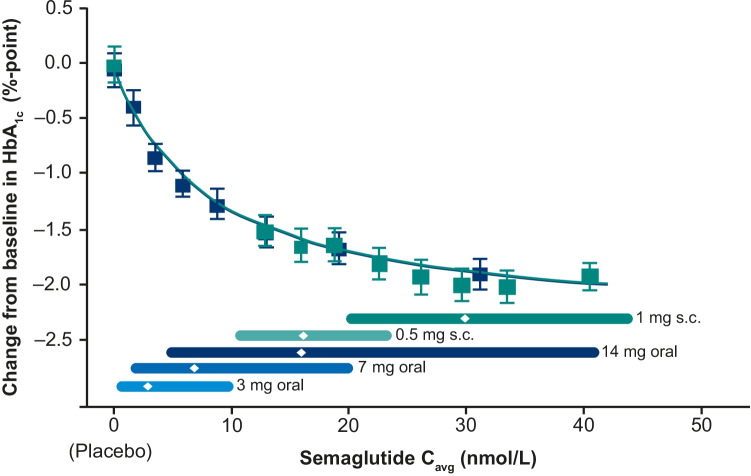
Change from baseline in HbA_1c_ in a propensity-score-matched population [94]. The plot shows the mean change and 95% CIs in HbA_1c_ from baseline to Week 26 for the PIONEER program (including PIONEER 1, 2, 3, 5, 8, and 9) and Week 30 for the SUSTAIN program (SUSTAIN 1, 2, and 3; SUSTAIN-Japan). Exposure is presented as quantiles of C_avg_ for semaglutide and one quantile for placebo (at C_avg_ of 0 nmol/L). The fitted solid line represents model-derived relations for each program. The horizontal lines along the x-axes represent medians and 90% exposure ranges; median exposure is represented by a diamond. Datasets were propensity-matched due to differences in demographics between the PIONEER and SUSTAIN programs. Blue represents PIONEER, green represents SUSTAIN. Reprinted from: Overgaard et al. Cell Rep Med. 2021;2(9):100387. https://doi.org/10.1016/j.xcrm.2021.100387. ^©^2021 The Author(s). With permission from Elsevier (CC BY-NC-ND 4.0). *C*_*avg*_ average plasma concentration, *CI* confidence interval, *HbA*_*1c*_ glycated hemoglobin, *s.c.* subcutaneous

Propensity-matching analysis using data from the SUSTAIN (subcutaneous semaglutide) and PIONEER (oral semaglutide) programs demonstrated that exposure–response relationships for efficacy and safety were consistent between oral and subcutaneous semaglutide (Fig. 5) [94].

### Cardiovascular outcomes

PIONEER 6 was an event-driven, preapproval CVOT in patients with T2D and high CV risk with follow-up until accrual of ≥ 122 MACE. The primary outcome – MACE – occurred in 3.8% of patients receiving oral semaglutide (61/1,591) versus 4.8% receiving placebo (76/1,592), with a 21% difference in risk (hazard ratio [HR]: 0.79; p < 0.001 for non-inferiority) [79]. In SUSTAIN 6 – the preapproval CVOT for subcutaneous semaglutide – the rate of CV death, nonfatal myocardial infarction, or nonfatal stroke was significantly lower among patients receiving semaglutide (6.6% [108/1,648]) than among those receiving placebo (8.9% [146/1,649]; HR: 0.74; p < 0.001 for non-inferiority; p = 0.02 for superiority, not prespecified) [30]. The similarity of the HRs in PIONEER 6 and SUSTAIN 6 suggests that the CV effect of semaglutide is independent of the administration route [30, 79]. Furthermore, post-hoc analyses of SUSTAIN 6 and PIONEER 6 combined revealed a HR of 0.76 for the effect of semaglutide versus placebo on overall MACE, driven mainly by the effect on nonfatal stroke [95]. A recent meta-analysis has subsequently confirmed a clear class effect of GLP-1RAs in reducing MACE in patients with T2D [96].

It is important to note that PIONEER 6 was designed to rule out an excess risk of CV events with oral semaglutide versus placebo; it therefore enrolled fewer patients and was shorter in duration than would be required to show safety in a post-approval setting, and was not powered to show superiority [79, 95]. The SOUL study is an ongoing CVOT in patients with T2D that will compare the effects of oral semaglutide versus placebo on the occurrence of MACE [97].

## From trials to translation: oral semaglutide in practice

As per the ADA and AACE/ACE guidance, a patient-centered approach should be adopted when treating individuals with T2D, taking into consideration efficacy in addition to patient-specific factors and preferences [15, 17]. If treatment intensification is required to improve glycemic control, oral medications (e.g., SGLT2is, DPP-4 inhibitors, sulfonylureas, or thiazolidinediones) and injectables (GLP-1RAs and insulins) can be considered, with many options for combining these therapies to step up treatment as needed to improve glycemic control [15, 17]. Patient-centered factors include comorbidities (established ASCVD or indicators of high-risk established kidney disease, or heart failure), the presence of a compelling need to minimize hypoglycemia, the need to promote weight loss or minimize weight gain, and cost [15, 17]. In cases where there is a need to minimize hypoglycemia or support weight loss, GLP-1RAs may be preferred to insulin [15, 17]. In patients with established ASCVD, clinicians should consider using GLP-1RAs or SGLT2is with proven CVD benefit, independent of baseline HbA_1c_, individualized A1C target, or metformin use [15, 17]. At present, this recommendation includes subcutaneous semaglutide but does not yet include oral semaglutide [15, 17].

Oral semaglutide was approved in 2019 as an adjunct to diet and exercise to improve glycemic control in adults with T2D [24]. As the first orally administered GLP-1RA, oral semaglutide broadens the options available to patients and healthcare providers who may have expressed reluctance to use injectables [3, 37–39].

When using oral semaglutide, it is important to educate patients about potential GI symptoms that may occur, including nausea, abdominal pain, diarrhea, decreased appetite, vomiting, and constipation. These are typically mild to moderate in intensity, most often occur during dose escalation [24, 38, 39, 61, 62, 76–82], and most likely reflect the mechanism of action of semaglutide in terms of delaying gastric emptying and increasing satiety via a central action [56]. To minimize potential GI side effects and support tolerability, oral semaglutide should be initiated at 3 mg once daily for 30 days; the dose is then increased to 7 mg once daily for glycemic control [24, 70]. Following at least 30 days of 7 mg once daily, the dose may be increased to 14 mg once daily if additional glycemic control is required [24].

When prescribing oral semaglutide, patients should be advised to swallow the tablets whole with water (120 mL) at least 30 min prior to the first ingestion of food, beverage, or any other oral medications of the day [24]. Patients treated with oral semaglutide (14 mg daily) can be transitioned to subcutaneous semaglutide (0.5 mg once weekly) the day after their last dose of oral semaglutide [24]. Patients treated with subcutaneous semaglutide (0.5 mg once weekly) can be transitioned to oral semaglutide (7 mg or 14 mg once daily) [24], and can be initiated up to 7 days after their last injection. There is no dose of oral semaglutide equivalent to subcutaneous semaglutide 1 mg [24].

## Conclusions

Semaglutide is the first GLP-1RA to be approved in an oral formulation for the treatment of T2D and illustrates how the transcellular carrier-based permeation enhancer SNAC can overcome the barriers to oral administration. Unlike other permeation enhancers, SNAC does not require a protective enteric coating and has been extensively tested with a range of molecules and assigned GRAS by the US FDA. Co-formulation of semaglutide with SNAC enables localized absorption of semaglutide in the stomach without affecting the absorption of other molecules. Model-based analyses suggest that semaglutide has a similar pharmacokinetic profile once absorbed, regardless of whether it is administered via the subcutaneous or oral route. Clinical studies have shown oral semaglutide to have superior efficacy to placebo and to a number of oral and injectable active comparators representative of common drug classes (DPP-4 inhibitors, other GLP-1RAs, SGLT2is), with a safety and tolerability profile consistent with the GLP-1RA class.

The development of an oral semaglutide formulation with a similar exposure−response relationship to the well-established injectable formulation of semaglutide is a significant milestone in the treatment of T2D and in overcoming the barriers to oral peptide therapy. The availability of the first oral GLP-1RA not only expands the repertoire of highly effective treatments for patients with T2D but may also mark the beginning of a new era for oral peptides.

## Data Availability

Data sharing is not applicable to this article as no datasets were generated or analyzed during the current study.

## Abbreviations

AACE/ACE: American Association of Clinical Endocrinologists/American College of Endocrinology
ADA: American Diabetes Association
ASCVD: Atherosclerotic cardiovascular disease
AUC: Area under the curve
C_avg_: Average plasma concentration
CI: Confidence interval
C_max_: Maximum concentration
CV: Cardiovascular
CVD: Cardiovascular disease
CVOT: Cardiovascular outcomes trial
DPP-4: Dipeptidyl peptidase-4
empa: empagliflozin
ETD: Estimated treatment difference
FDA: Food and Drug Administration
GI: Gastrointestinal
GLP-1: Glucagon-like peptide-1
GLP-1RA: Glucagon-like peptide-1 receptor agonist
GRAS: Generally regarded as safe
HbA_1c_: Glycated hemoglobin
HR: Hazard ratio
imp.: impairment
lira: liraglutide
MACE: Major adverse cardiovascular events
met: metformin
OAD: Oral antidiabetes drug
pbo: placebo
s.c.: subcutaneous
sema: semaglutide
SGLT2i: Sodium-glucose co-transporter-2 inhibitor
sita: sitagliptin
SNAC: Sodium N-(8-[2-hydroxybenzoyl]amino)caprylate
SU: Sulfonylurea
t_1/2_: Terminal half-life
T2D: Type 2 diabetes
T_max_: Time to maximum concentration
